# Skin Markers of Premature Ageing in Patients with COPD: Results Form COSYCONET

**DOI:** 10.3390/jcm13226972

**Published:** 2024-11-19

**Authors:** Thomas Melzer, Veronika Graf, Angelika Kronseder, Stefan Karrasch, Martina Kerschner, Claus F. Vogelmeier, Robert Bals, Peter Alter, Henrik Watz, Sebastian Fähndrich, Jürgen Behr, Benjamin Waschki, Franziska Christina Trudzinski, Rudolf A. Jörres, Kathrin Kahnert

**Affiliations:** 1 Department of Medicine V, LMU University Hospital, LMU Munich, Comprehensive Pneumology Center, Member of the German Center for Lung Research (DZL), 80336 Munich, Germany; veronika_graf@psych.mpg.de (V.G.); juergen.behr@med.uni-muenchen.de (J.B.); kathrin.kahnert@med.uni-muenchen.de (K.K.); 2 Max Planck Institute of Psychiatry, 80804 Munich, Germany; 3 Institute and Outpatient Clinic for Occupational, Social and Environmental Medicine, University Hospital, LMU Munich, Comprehensive Pneumology Center Munich (CPC-M), German Center for Lung Research (DZL), 35392 Munich, Germany; angelika.kronseder@med.uni-muenchen.de (A.K.); stefan.karrasch@med.uni-muenchen.de (S.K.); rudolf.joerres@med.uni-muenchen.de (R.A.J.); 4 Division of Cosmetic Science, Department of Chemistry, University of Hamburg, 20148 Hamburg, Germany; martina.kerscher@uni-hamburg.de; 5 Department of Medicine, Pulmonary and Critical Care Medicine, University Medical Center Giessen and Marburg, Philipps-University, German Center for Lung Research (DZL), 35392 Marburg, Germany; clausfranz.vogelmeier@uk-gm.de; 6 Department of Internal Medicine V—Pulmonology, Allergology, Critical Care Care Medicine, Saarland University Hospital, 66421 Homburg, Germany; robert.bals@uks.eu; 7 Department of Medicine, Pulmonary and Critical Care Medicine, Philipps University of Marburg (UMR), 35037 Marburg, Germany; alter@uni-marburg.de; 8 Pulmonary Research Institute, Lungen Clinic Grosshansdorf, 22927 Grosshansdorf, Germany; h.watz@pulmoresearch.de; 9 Airway Research Center North (ARCN), German Center for Lung Research (DZL), Woehrendamm 80, 22927 Grosshansdorf, Germany; b.waschki@kh-itzehoe.de; 10 Department of Pneumology, University Medical Centre Freiburg, 79106 Freiburg, Germany; sebastian.faehndrich@uniklinik-freiburg.de; 11 Department of Pneumology, Itzehoe Hospital, 25524 Itzehoe, Germany; 12 Department of Pneumology and Critical Care Medicine, Thoraxklinik, Translational Lung Research Center Heidelberg (TLRC-H), German Center for Lung Research (DZL), University of Heidelberg, 69117 Heidelberg, Germany; franziska.trudzinski@med.uni-heidelberg.de; 13 MediCenter Germering, 82110 Germering, Germany

**Keywords:** age-related diseases, exposome, COPD, emphysema, skin wrinkles

## Abstract

**Background:** Chronic obstructive pulmonary disease (COPD) is commonly associated with ageing, with the prevalence and severity increasing by age. Smoking-induced premature ageing is thought to contribute to COPD, particularly lung emphysema. This study aimed to explore the relationship between lung function impairment and skin texture, as a marker of biological or premature ageing, in COPD patients. **Methods:** A subcohort from the COSYCONET COPD-study was analyzed, where skin-relief replicas of the eye’s outer corner and mid-lower inner arm were collected, along with semi-quantitative facial photographs. We examined the correlation between skin parameters and lung function, particularly the diffusing capacity (TLCO) as an indicator of emphysema. **Results:** Among 46 COPD patients (69 ± 8 years, 52% female), skin texture from the inner forearm, but not from the eye corner, was significantly associated with TLCO% predicted, with a higher skin roughness correlating with a lower TLCO (*p* = 0.015). This relationship persisted after adjusting for age, BMI, sex, pack years, and smoking status. No significant associations were found with facial photographs. **Conclusions:** These findings suggest that systemic ageing, reflected in inner arm skin texture, is linked to lung emphysema. Skin ageing markers may be valuable in future interventional studies involving anti-ageing treatments.

## 1. Introduction

Chronic obstructive pulmonary disease (COPD) is often associated with pulmonary and extra-pulmonary comorbidities [1], among which cardiac comorbidities are particularly important [2]. In addition to shared genetic risk factors, the occurrence of comorbidities might be explained by the commonality of risk factors. The most significant extrinsic risk factor is smoking, which induces or promotes cellular changes similar to those observed with biological ageing. Typical manifestations are skin ageing, which is often particularly noticeable in the face, but also cardiovascular and cerebrovascular diseases, the frequency of which also increases with age [3]. Systemic inflammation may be a mediator for various COPD-associated diseases as well as ageing, but it has not been conclusively clarified to which extent it is a causal factor [4].

With regard to mortality, specifically cardiac mortality in smokers and former smokers, lung function has been identified as an independent predictor [5] that might be superior to established risk factors for cardiovascular diseases such as elevated BMI, serum cholesterol, or blood pressure [3]. Lung function may reflect structural processes associated with the biological ageing of the lungs [3,6]. To evaluate the effects of biological ageing on the whole lung, we assessed different lung function parameters representing airway obstruction, hyperinflation, and gas exchange [6].

Biological ageing encompasses a variety of cellular, molecular, and structural changes. It can also occur prematurely as a result of external factors. This process is called “induced ageing” and can be elicited by cigarette smoking [3]. Previous studies have already shown an association between premature ageing and COPD development. The aim of the present study was to investigate this concept via the assumption that induced ageing has a systemic dimension and can thus be identified in several organs. One of the most easily accessible organs is the skin. We therefore studied the relationship between skin ageing and parameters of lung function impairment, as two potential markers of ageing [7,8,9]. To address this question a local sub-cohort of the German COPD cohort COPD and Systemic Consequences–Comorbidities Network (COSYCONET) was used [10].

## 2. Materials and Methods

### 2.1. Study Population

The prospective, multi-center German COPD cohort study COSYCONET started in 2010 in 31 study centers all over Germany (see also Supplementary Materials and [10]). Its aim was to assess the interaction of lung disease, comorbidities, and systemic inflammation [10]. N = 46 patients of the Munich study center who participated in the COSYCONET follow-up visits 4 and 5 (36 and 54 months after recruitment) were asked to participate in the present sub-study. For the additional investigations in this sub-study, ethics approval was provided by the local ethical committee and an additional written informed consent form was applied.

### 2.2. Ethics Approval and Consent to Participate in COSYCONET

The study was conducted in accordance with the amended Declaration of Helsinki. Ethical review and approval were principally conducted by the central ethical committee in Marburg, Germany (Ethikkommission FB Medizin Marburg). Local ethical commitees stated their agreement at each study site: Bad Reichenhall (Ethikkommission bayerische Landesärztekammer); Berlin (Ethikkommission Ärztekammer Berlin); Bochum (Ethikkommission Medizinische Fakultät der RUB); Borstel (Ethikkommission Universität Lübeck); Coswig (Ethikkommission TU Dresden); Donaustauf (Ethikkommission Universitätsklinikum Regensburg); Essen (Ethikkommission Medizinische Fakultät Duisburg-Essen); Gießen (Ethikkommission Fachbereich Medizin); Greifswald (Ethikkommission Universitätsmedizin Greifswald); Großhansdorf (Ethikkommission Ärztekammer Schleswig–Holstein); Hamburg (Ethikkommission Ärztekammer Hamburg); MHH Hannover/Coppenbrügge (MHH Ethikkommission); Heidelberg Thorax-/Uniklinik (Ethikkommission Universität Heidelberg); Homburg (Ethikkommission Saarbrücken); Immenhausen (Ethikkommission Landesärztekammer Hessen); Kiel (Ethikkommission Christian-Albrechts-Universität zu Kiel); Leipzig (Ethikkommission Universität Leipzig); Löwenstein (Ethikkommission Landesärztekammer Baden-Württemberg); Mainz (Ethikkommission Landesärztekammer Rheinland-Pfalz); München LMU/Gauting (Ethikkommission Klinikum Universität München); Nürnberg (Ethikkommission Friedrich-Alexander-Universität Erlangen Nürnberg); Rostock (Ethikkommission Universität Rostock); Berchtesgadener Land (Ethikkommission Land Salzburg); Schmallenberg (Ethikkommission Ärztekammer Westfalen-Lippe); Solingen (Ethikkommission Universität Witten-Herdecke); Ulm (Ethikkommission Universität Ulm); and Würzburg (Ethikkommission Universität Würzburg]. Written informed consent has been obtained from all participants.

### 2.3. Assessments

Inclusion and exclusion criteria for recruitment, the study protocol, and the assessments have been published previously [10] and are given in the Supplementary materials. Following the COSYCONET study protocol, lung function analysis comprising spirometry, bodyplethysmography, and the determination of diffusing capacity for carbon monoxide (CO) were performed following established recommendations [10]. Reference values from the Global Lung Function Initiative (GLI) or European Coal and Steel Community (ECSC) were used [11]. Details of the COSYCONET study protocol and the respective standard operating procedures have been published previously [10]. In addition to the standard COSYCONET workup, skin relief replicas from the face and mid-lower arm were taken. Portrait photographs were used for a visual assessment of the facial skin. The analysis of these samples served to determine skin ageing. Regarding lung ageing, we selected as primary parameter diffusing capacity for CO which is known to be related to the degree of emphysema [12,13], while emphysema is associated with induced ageing [14]. As secondary parameters, airway obstruction in terms of FEV1% predicted and air trapping in terms of RV/TLC were chosen, both of which are also related to ageing [3,6,15].

### 2.4. Skin Relief Replicas

**Figure 1:**
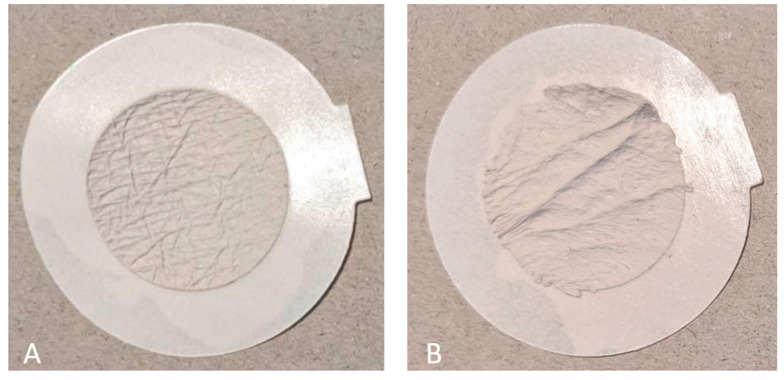
(**A**,**B**) Examples of skin relief replicas obtained from a study participant. Each replica is localized on a larger-sized carrier ring which is needed for handling and stability of the casts. The replica of the forearm inner side in (**A**) can be easily discriminated from the right eye’s outside corner in (**B**) due to more prominent deep skin wrinkles.

The SILFLO^®^ casting kit (Monaderm, Monte Carlo, Monaco) was deployed to obtain two skin relief replicas from each participant, one from the right eye’s outside corner and one from the inner side of the right the mid-lower arm. Patients were asked to lie down on an examination couch and place their right the mid-lower arm with the inside facing upwards. First, a self-adhesive ring was applied to the described areas; then, the impression material and the catalyst were mixed according to the manufacturer’s instructions and applied thinly and evenly. Approximately 5 min later, after drying, the impressions could be removed and stored in a refrigerator until analysis. Examples of skin relief replicas can be seen in Figure 1A,B.

For the quantitative analyses of the casts, we used the optical 3-dimensional measuring device Primos compact (LMI Technologies GmbH, Teltow, Germany). Skin relief replicas were scanned to achieve 3D-images in which the vertical shape was color-coded.

In order to analyze wrinkles as depressions in the skin, the image was digitally inverted. The program function ‘invalid’ was used to fill potential minor gaps in the 3D images. Although gaps occurred in only few images, the function was applied to all datasets to avoid bias. Furthermore, several digital filters as part of the software package were used. The Gaussian filter 3 × 3 enabled a slight smoothing to reduce excessive gradients that would interfere with the analysis. A robust high-pass filter was used to remove the bending of the entire surface from the measurement data. This was the best way to ensure that the evaluation took into account only the local surface relief and not the overall curvature of the mid-lower arm or eye rim.

The final evaluation of the two impression surfaces by software had to be carried out differently. Wrinkle analysis was performed to analyze the wrinkle structure of the right eye’s outside corner, while various roughness parameters were computed to describe the surface of the mid-lower arm. The most important parameters used in this study and their abbreviations are described in Table S1 (Supplementary Materials).

Photographic images of the skin and their evaluation:

In addition to the skin relief replicas, each participant underwent portrait photography (Cybershot DSC-H20 camera, Sony, Minato, Japan) in P-automatic mode with additional indirect lighting and a tripod. All photos were taken in standardized frontal and two half-profile perspectives. For better orientation in the evaluation of the photographs, 5 × 5 mm grids were stuck to the patient’s cheeks. The portraits were qualitatively analyzed by three independent examinators and the results were averaged. Specifically, the nasolabial (NL) and periorbital (PO, crow’s foot region) skin wrinkles were categorized into severity grades from 0–4 (0 = no skin wrinkles, 4 = severe skin wrinkles), a procedure in line with previous work in this field [16,17,18,19,20].

### 2.5. Statistical Analyses

Mean values (MWs) and standard deviations (SDs) were calculated if the distribution of the values justified these operations. Medians, maxima, and minima were also calculated. The unpaired t-test or the Mann–Whitney U-test was used to compare two groups, depending on the distribution, and a one-way analysis of variance (ANOVA) was used to compare more than two groups. Simple or multiple linear regression analysis was used to determine associations between continuous variables. Correlation coefficients were calculated according to Pearson or Spearman in order to visualize the strength of the correlation between two variables. Furthermore, the correlation structure of individual datasets was determined using factor analyses (principal component method with Varimax rotation) in order to identify single representative parameters. For inferential statistics, *p*-values < 0.05 were considered as statistically significant. The software packages SPSS Version 25 (IBM Corporation, Armonk, NJ, USA) as well as Prism 6.01 (Graph Pad Software Inc., San Diego, CA, USA) were used for statistical analyses; see also Table S2 (Supplementary Materials).

## 3. Results

### 3.1. Study Population and Lung Function Analyses

**Figure 2:**
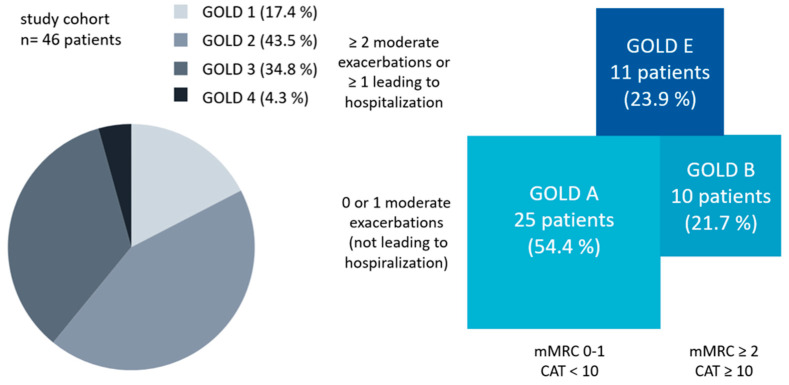
The study cohort according to the GOLD 2023 report. The area ratios in the right part of the graph correspond to the percentage distribution of the study cohort.

The basic characteristics of the participants are shown in Table 1 and Figure 2. The study population comprised 46 patients (52.2% females) with a median age of 69 ± 8 years. Most patients had a moderate airway obstruction according to GOLD grades 2 and 3 (78.3% of all patients). The distribution of GOLD grades and groups is given in Figure 2.

### 3.2. Analyses of Skin Relief Replicas

Skin relief replicas from the right eye’s outside corner were analyzed by wrinkle analysis (Table S3, Supplementary Materials), and those of the inner side of the mid-lower arm with regard to roughness parameters (Table S4, Supplementary Materials). The reason was that wrinkles next to the right eye’s outside corner were too deep to calculate valid roughness parameters, whereas the wrinkles of the mid-lower arm were too flat for wrinkle analysis. There were significant correlations between parameters for either those of the right eye’s outside corner or those of the inner side of the mid-lower arm , while correlations between parameters of both regions were absent (Table 2). In the same manner, the roughness parameters of the mid-lower arm were analyzed, with resulting eigenvalues of 6.956, 1.759, and 1.185 that explained 90.0% of variance. The rotated matrix of loadings is shown in Table 3, again indicating that the different indices could be fairly clearly attributed to one of the three factors. Summarizing the results, the skin parameters of the eye and the mid-lower arm represented two and three degrees of freedom, respectively, and showed a distinctive pattern of correlation.

### 3.3. Correlation of Skin Relief Replicas and Lung Function Analyses

The TLCO% predicted was the lung function variable of greatest interest as a surrogate marker of the biological ageing of the lung. It was correlated with the skin parameters Sa, Sq, Smax, Sz, and Sp of the mid-lower arm (*p* < 0.05 each). Choosing the skin parameters of the right eye’s outside corner, no significant correlation with TLCO% predicted was observed. There were no significant correlations with RV/TLC and FEV1% predicted (*p* > 0.05 each) for any of the skin parameters. Based on the correlation coefficients with TLCO and the degree of separation of loadings between factors as shown in Table 3, we selected Sp as the representative parameter of the mid-lower arm skin characteristics.

A simple bivariate regression analysis showed that Sp and TLCO% predicted were significantly (*p* = 0.012) related to each other. First skin relief is dependent on age. Second, there could be an effect of BMI based on two mechanisms. A low BMI, especially cachexia, is associated with both a higher prevalence of lung emphysema [21,22,23] and accentuated skin wrinkling [24,25], while a higher BMI might reduce the texture due to the higher tension in the skin. To account for this, we included BMI as a continuous variable in the regression analysis. Further covariates of interest were age, sex, smoking status and pack years, all of which might influence skin ageing.

When including these covariates, a significant (*p* = 0.015) association between Sp and TLCO% predicted was still found (Table 4), while none of the other variables was identified as significant. It should be noted that the other skin parameters (Sa, Sq, Smax, Sz, Sv, Sk, and Sdr) that showed significant correlations with Sp and were assigned to the same dimension in the factor analysis (Table 3) were also significantly associated with TLCO% predicted in analogous linear regression analyses (*p* < 0.05 each), demonstrating that Sp covered a generic feature of skin texture. To account for the possibility that the association with the BMI could be non-linear, we defined an indicator variable of lower versus higher BMI, with a cut-off value of 25 kg/m^2^. When repeating the analysis with this binary variable, the relationship of Sp to TLCO% predicted remained (*p* = 0.005), while, at the same time, the two BMI categories were also significant (*p* = 0.039). These sensitivity analyses underlined the robustness of the association between skin parameters and diffusing capacity.

### 3.4. Skin Analyses by Portrait Photography

Wrinkles in the nasolabial region but not in the periorbital region correlated significantly (*p* < 0.05) with the inner-arm parameter Sp that was chosen for the analysis of skin replicas, indicating the consistency of the data. The periorbital wrinkles were not significantly related to TLCO% predicted, taking into account the covariates included for Sp. In addition, the wrinkles in the nasolabial region were not significantly related to TLCO (% predicted), while there was only a tendency (*p* = 0.066), in contrast to BMI (*p* = 0.010).

## 4. Discussion

In the present study, we found a statistically significant relationship between the TLCO and skin roughness parameters of the mid-lower arm, as markers of ageing. The association was robust even after adjusting for major factors potentially influencing skin texture such as BMI, age, and smoking status. This is of interest as the diffusing capacity is closely related to lung emphysema [13] and lung emphysema is often thought to be linked to lung ageing [3], even to the extent that potential anti-ageing compounds are associated with a reduced decline in lung diffusing capacity over time, such as metformin [13,26]. Both epidemiological and experimental data suggest that metformin might attenuate the progression of lung emphysema [26,27]. This fits into the view that antidiabetic agents may have beneficial effects on the lung beyond blood glucose control, probably to their anti-inflammatory and antioxidant potential [28,29]. Besides metformin, SGLT2-inhibitors have been shown to exert beneficial effects, even on exacerbations, in COPD, despite the fact that their target transporter is not expressed in the lung [29,30,31]. This indicates that systemic factors play a role regarding lung disease, and such systemic factors might comprise ageing as reflected in different organs including the skin.

Previous studies have shown that facial wrinkling is associated with the FEV1 decline and emphysema extent in CT-scans [32]. However, pulmonary diffusion capacity as a functional marker of lung emphysema has not been analyzed yet. Remarkably, in our study, there was no association of spirometric lung function parameters with skin roughness indicating a specific effect on the phenotype emphysema. As vascular diseases are also thought to be associated with ageing [28] and vascular alterations play a major role in the initiation and progression of emphysema [33,34], the pattern observed in the present study is consistent and in line with previous findings [27]. None of the lung function parameters including TLCO% predicted was associated with skin wrinkling parameters of the eye region, as an alternative marker of skin ageing. This might be explained by the fact that the eye region is more affected by external factors such as sunlight and other exposures compared to the inner arm, rendering the respective parameters better indicators of systemic ageing and thus more closely related to lung ageing.

The present study was conducted in a local sub-cohort of COSYCONET. Its baseline characteristics showed a mean age of 69 years and typical functional patterns of GOLD grades 2 and 3 [10,35]. Based on a functional emphysema score defined previously [13], the presence of lung emphysema in this population could be assumed in about 32% of patients, which underlines that our cohort was suitable for the aim of the present analysis.

In order to assess skin characteristics, we followed two different approaches. Portrait photography of patients with subsequent evaluation by experts has been previously used for investigations related to biological ageing in COPD, as an easy and non-invasive way of data acquisition [32]. This method may be criticized due to its semi-quantitative nature. In contrast, the second method involved casts of the skin wrinkle relief [36] and numeric analyses of skin folds. These analyses were performed using a specific camera system and software [37,38,39] delivering detailed, objective, and quantitative skin parameters that could be superior to photographs. This, however, is not a priori guaranteed, and we thus employed both methods.

Many factors have been described to influence skin ageing, especially exposure to ultraviolet radiation (UV) [40]. The human midface is, most of the time, uncovered from radiation and affected by mimical muscles [41], thus wrinkle assessments via portraits might be confounded when aiming to determine systemic ageing [42]. For example, some investigators assume that cigarette smoke could directly induce damage in facial skin by surface contact [43,44,45,46] and thus alter the results of measurements. To identify effects mainly driven by endogenous, and not exogenous, ageing, we chose an additional skin region that could be considered less affected by external factors. We therefore chose the mid-lower arm as the site for skin relief replicas, in addition to the right eye’s outside corner, assuming that this area would be affected mostly by systemic, endogenous factors of biological ageing driven by smoking, that would also lead to emphysema-related ageing and a reduced diffusing capacity. Being often covered by clothing, the mid-lower arm’s wrinkle structure might be less influenced by radiation and other factors [37].

The skin relief replicas yielded a broad panel of parameters compared to the few portrait-based scores. This raised the question of the relationship between parameters, in order to identify the most appropriate ones. For this purpose, we used explorative factor analyses. These showed, for the eye’s corner, two factors and, for the mid-lower arm, three factors, i.e., essentially, sets into which the different parameters could be summarized based on their mutual correlation. From these sets, we chose representative parameters in order to assess their potential relationship with lung function, specifically with the diffusing capacity.

The regression analyses did not indicate any association between the skin relief parameters of the right eye’s corner and lung function indices. In contrast, there were associations for the skin roughness parameters of the mid-lower arm. This is shown for the parameter Sp representing the elevation of the highest skin profile tips. In bivariate, unadjusted analyses, Sp was significantly related to the diffusing capacity. As it is obvious that skin texture can be affected by age, sex, BMI, current smoking status, and accumulated pack years, we then added these variables as confounders demonstrating a robust relationship between Sp and TLCO% predicted.

It has been discussed that, especially, overweight might induce stretching effects on the skin, resulting in less common and prominent wrinkles [24,25]. Conversely, low weight, which is not uncommon in COPD patients, should increase the wrinkling. The mean BMI of our study cohort of 27.2 kg/m^2^ was similar to the mean BMI of the total COSYCONET cohort [10], indicating that slight overweight is common in COPD patients. Low values of BMI may be caused by disease-induced weight loss [47]. Especially for pulmonary cachexia, it could be assumed, on the one hand, that skin wrinkling is driven by the loss of subcutaneous fat and muscle mass [48] so that the tightening effect of the subcutis is eliminated. On the other hand, the epidermis could be directly affected. As it is generally known that cachexia includes the occurrence of an extensive protein turnover resulting in mainly degradation [48], the previously described loss of elastin in COPD could be a major component of ageing in patients with a low BMI [49]. Based on these considerations, we performed a separate analysis in patients with a BMI ≥ 25 kg/m^2^ or less than < 5 kg/m^2^. The range of TLCO values covered by the two BMI groups was similar, and the group with a BMI ≥ 25 kg/m^2^ also included patients with a low diffusing capacity. The association between Sp and TLCO% predicted was still present in the patients with a BMI ≥ 25 kg/m^2^ but not in that with a lower BMI, probably due to the causes indicated above.

Remarkably, there were associations between parameters of the inner arm and nasolabial folds, but not periorbital wrinkles. Consistent with that, there was at least a tendency for nasolabial folds being associated with the diffusing capacity when taking into account confounders like age, BMI, and smoking status. The superiority of the inner arm parameters might not only be due to the fact that they were quantitative, objective parameters in contrast to the semi-quantitative rating of the photographs but also due to the fact that nasolabial folds are affected by external factors similar to periorbital wrinkles. In any case, our study demonstrates that skin replicas of less exposed areas are more suitable for assessing systemic ageing than photographs.

The results underline the hypothesis of premature ageing in COPD patients, which is also reflected in the skin. Future research could therefore focus on the effect of potential systemic anti-ageing substances on the lungs and other organ manifestations including the skin. In addition, more sophisticated skin analyses, especially as part of an interdisciplinary pneumological–dermatological research approach, could help to better investigate premature skin ageing in COPD patients to improve clinical status, quality of life, and, at the same time, aesthetic appearance.

Irrespective of the direct link between skin ageing parameters and emphysema, this study also emphasizes that long-term cigarette smoking causes structural changes in terms of premature skin ageing. These are associated with a loss of elasticity and deeper wrinkles. Thus, our findings supplement the existing literature that already describes premature skin ageing and the higher risk of pigment spots, sallow coloring, squamous cell carcinoma, and impaired wound healing in smokers [50,51,52]. Additionally, a risk for basal cell carcinoma has been reported [53]. This emphasizes the urgent need for smoking cessation in patients with COPD from a multitude of perspectives [54].

### Limitations

Within the COSYCONET sub-cohort at the study center of the LMU Munich, only 46 patients could be recruited for the present study. This resulted in a limited statistical power. Nevertheless, a robust relationship between the TLCO% predicted and skin roughness parameters was found. This supported the hypothesis of ageing as a systemic factor in COPD that had parallel effects on the lung and the skin, provided the appropriate region was chosen. It certainly would have been of interest to include other regions that might be assumed as having a low exposure to external factors, such as the back or the buttocks, but this could not be realized within the present setting.

## 5. Conclusions

In patients with COPD, we observed an association between the skin roughness of the mid-lower inner arm, as a hypothetical marker of skin ageing, and the diffusing capacity of the lung, as a hypothetical marker of lung ageing related to the development of emphysema. This association was robust against other influencing factors such as age, sex, smoking status, and BMI. Therefore, our findings support the hypothesis that systemic ageing plays a role in the development of COPD, specifically lung emphysema. This is consistent with recent data on the effects of potential anti-ageing compounds. In order to cover the full set of causal factors in COPD, the assessment of markers of ageing, either systemic or organ-specific, might thus be of great interest particularly in interventional studies that involve compounds, for which anti-ageing effects either have been shown or are assumed.

## Figures and Tables

**Table 1:** Patient characteristics of the study population.

Variables	Mean ± SD
Sex (m/f) (numbers) (%)	22/24 (47.8/52.2)
Age (years)	69 ± 8
Body height (m)	1.68 ± 0.11
Body weight (kg)	76.9 ± 14.6
BMI (kg/m^2^)	27.2 ± 3.8
GOLD 1/2/3/4 [n]	8/20/16/2
GOLD 1/2/3/4 [%]	17.4/43.5/34.8/4.3
GOLD A/B/E [n]	25/10/11
GOLD A/B/E [%]	54.4/21.7/23.9
FEV_1_ (% predicted)	59.1 ± 22.8
FVC (% predicted)	80.7 ± 17.8
FEV_1_/FVC	0.55 ± 0.13
TLCO (% predicted)	70.5 ± 22.5
KCO (% predicted)	72.5 ± 20.2
Pack years	44.6 ± 34.8
Packs per day	1.7 ± 1.3
6-min-walking-distance (m)	448.8 ± 84.0
CAT	17.1 ± 6.9
mMRC	0.59 ± 0.58
RV (% predicted)	166.9 ± 50.7
RV/TLC (% predicted)	54.9 ± 10.8
ITGV (% predicted)	140.8 ± 41.6

(n = 46). Numbers are shown for categorial variables, and, for continuous variables, mean values and standard deviations. FEV1 = forced expiratory volume in 1 s, FVC = forced vital capacity, TLCO = transfer factor for carbon monoxide, KCO = transfer coefficient of carbon monoxide, CAT = COPD assessment test, mMRC = modified Medical Council Research Dyspnea Scale, RV = residual volume, TLC = total lung capacity, ITGV = intrathoracic gas volume. GOLD groups A/B/E were based on the current criteria of the GOLD recommendations 2023. According to this recommendation, the former GOLD groups C and D are combined as GOLD group E.

**Table 2:** Principal component analysis of right eye’s outside corner’s parameters.

	Factor	
	1	2
mean wrinkle depth	**0.962**	0.060
deepest wrinkle	**0.868**	0.111
wrinkle count	0.039	**0.947**
wrinkle volume	**0.930**	0.293
wrinkle surface	0.610	**0.716**
mean wrinkle form factor	0.354	−0.657
wrinkle length	0.445	**0.802**
mean depth of largest wrinkle	**0.902**	0.081
maximum depth of largest wrinkle	**0.875**	−0.111

Loadings are indicated to the two dimensions with eigenvalues 5.207 (1) and 2.220 (2) explaining most variance. Loadings exceeding an amount of 0.7 are indicated in bold letters.

**Table 3:** Principal component analysis of inner forearm’s parameters.

	Factor		
	1	2	3
Sa	**0.897**	0.154	0.321
Sq	**0.906**	0.153	0.292
Smax	**0.971**	0.003	−0.090
Sz	**0.975**	0.021	−0.081
Sp	**0.877**	−0.051	−0.064
Sv	**−0.966**	−0.040	0.102
Sk	**0.891**	0.156	0.337
S	0.057	**0.942**	−0.076
Ssk	0.042	−0.013	**0.900**
PC	−0.051	**−0.923**	−0.081
Sdr	**0.843**	0.013	0.441

Loadings are indicated for the three factors with eigenvalues 6.956, 1.759, and 1.185 explaining 90.0% of variance. Loadings exceeding 0.7 are indicated in bold letters.

**Table 4:** Linear regression analyses to determine the confounding of the Sp.

	Non-Standardized Coefficients		Standardized Coefficients	t	*p*
	Regression Coefficient b	Standard Error	Beta		
(constant)	29.020	58.685		0.494	0.624
TLCO% predicted	−0.632	0.246	−0.427	−2.562	0.015 (*)
age (years)	0.713	0.609	0.183	1.172	0.249
sex (m/f)	6.124	10.469	0.095	0.585	0.562
BMI (kg/m^2^)	1.904	1.413	0.222	1.348	0.186
smoker (+/−)	2.353	12.491	0.030	0.188	0.852
pack years	−0.087	0.149	−0.095	−0.586	0.561

–TLCO (%predicted) relationship. The regression coefficients were calculated for Sp as dependent variable and TLCO% predicted, respectively, with potential confounders as predictors. TLCO% predicted was the only significant (*) predictor. t is the corresponding t-distributed evaluation variable and p the significance level.

## Data Availability

The data shown in this article may be partly or fully obtained from a third party and are not publicly available. The dataset underlying the work in this article is available upon request from the Competence Network Asthma and COPD (ASCONET, http://www.asconet.net/html/cosyconet/projects, accessed 17 November 2024).

## Supplementary Materials

The following supporting information can be downloaded at: https://www.mdpi.com/article/10.3390/jcm13226972/s1. Table S1: Skin relief analyses parameters; Table S2: Software; Table S3: Key statistic parameters calculated by wrinkle analysis of patients’ right eye’s outside corner; Table S4: Key statistic parameters calculated by determination of the degree of roughness of patients’ right forearm The COSYCONET COPD cohort study. The COSYCONET Study (ClinicalTrials.gov ID NCT01245933) started recruitment in 2010 via 31 study centers across Germany. Regarding the ethics approval, please see under declarations. The aim was to assess interactions between pulmonary disease and systemic comorbidities as part of the German Asthma and COPD Network (ASCONET). Key inclusion criteria were patients diagnosed with COPD according to GOLD criteria or chronic bronchitis at age of 40 years and older and the estimated availability for several study visits at baseline and follow-up. Individuals past relevant lung volume reduction, and with transplantation, severe exacerbations within 4 weeks before recruitment, lung malignancies, and impairments resulting in inabilities to walk or understand the project were excluded. Written informed consent was obtained by each participant after detailed information on the study and its investigation program was provided. The study program included, among others, demographic assessment, clinical history, comorbidities and medication, patient’s questionnaires, anthropometry, lung function analytics, blood gas analysis, collection of blood and urine samples, and cardiologic, sleep, and functional exercises. Some assessments were only conducted in selected study centers, e.g., in-protocol-CT/MRI lung imaging which was not performed at the Munich center. A full characterization of the whole German cohort and the 2741 patients recruited was published by Karch et. al. [10]; all questionnaires and SOPs can be found on www.asconet.net/html/cosyconet and have been extensively described.
