# Supplementary material for: Skin Markers of Premature Ageing in Patients with COPD: Results Form COSYCONET
Source: J Clin Med. 2024 Nov 19;13(22):6972. doi: 10.3390/jcm13226972 (PMC11595569; doi:10.3390/jcm13226972)
Supplement: Supplementary file 1 [file jcm-13-06972-s001.zip › jcm-3281595-supplementary.pdf]

Supplementary Materials

Table S1

Parameter abbr.	Parameter	Description
Sa	arithmetic roughness	arithmetic mean of the average of the amounts of the profile values of the roughness profile
Sq	mean square mean value	mean square mean value of the profile roughness values
Smax	maximum extension of the roughness profile	maximum extension of the roughness profile, which is calculated by the height difference from the highest peak to the lowest valley within the total measurement area
Sz	ten-point height	ten-point height and is the average of the amounts of the 5 highest peaks and the 5 lowest depressions of the total measuring area
St	maximum roughness depth	maximum roughness depth, meaning the greatest height difference within a single measuring area
Sp	highest profile tip	height of the highest profile tip within the total measuring area
Sv	deepest profile depression	height of the deepest profile depression within the total measuring area
Ssk	obliquity	obliquity measure calculated by the asymmetry of the amplitude of the amplitude density curve
Sla	local profile slope	derived figure by the arithmetic mean of the local profile slope in x- and y-direction
PC	peak count	calculated by the number of peaks in the total measuring area
S	average peak distance	average distance between the profile peaks in the roughness profile
Sk	roughness kernel	depth of the roughness kernel

Table S1: Skin relief analyses parameters

Table S2

Application	Software, Release	Publisher	Principal office
Data preparation	Microsoft Excel, Release 2105, Microsoft 365	Microsoft Corporation	Redmond, United States of America
Statistics	Prism 6.01	Graph Pad Software Inc.	San Diego, United States of America
Statistics	SPSS Statistics for Windows, Version 25	IBM Corporation	Armonk, United States of America
Literature Management	EndNote 20.6, BLD 17174	Clarivate Analytics	Philadelphia, United States of America
Manuscript preparation	Microsoft Word Release 2105, Microsoft 365	Microsoft Corporation	Redmond, United States of America

Table S2: Software

Table S3

	mean depth (µm)	deepest wrinkle	wrinkle count	wrinkle volume (mm^3)	wrinkle surface (mm^2)	mean wrinkle form factor	wrinkle length (mm)	mean depth of largest wrinkle (µm)	maximum depth of largest wrinkle (µm)
minimum	23.5	27	4	0.29	12.4	1.1	23	24	69
maximum	115.9	201	89	4.91	50.9	2.1	111	158	931
mean	51	84	31	1.99	37	1.6	67	64	276
SD	20.6	39	18	1.09	8.9	0.2	19	30	179
median	48.5	79	26	1.76	38.4	1.6	67	59	227

Table S3: Key statistic parameters calculated by wrinkle analysis of patients' right eye's outside corner using skin relief replicas and ODSCAD 6.3/PRIMOS 5.7 software.

Table S4

	Sa	Sq	Smax	Sz	St	Sp	Sv	Ssk	Sku	S
minimum	8.8	11.5	129.5	126.2	129.5	49.4	-297.1	-1.03	3.05	251.3
maximum	31.5	42.2	440.9	428.1	440.9	184.7	-80.1	0.01	5.71	763.5
mean	17.5	22.6	226.6	216.5	226.6	90.6	-136	-0,6	4.0	534.2
SD	6.3	8.0	75.61	72.93	75.61	32.14	46.74	0.24	0.61	102.76
median	15.8	20.4	206.1	191.2	206.1	77.9	-128.2	-0.61	3.93	520.7
	Sda	Sdq	Sla	Slq	PC	Sdr	Sk	Spk	Svk	
minimum	0.1144	0.2142	0.4685	0.3274	128.0	2.239	28.2	9.9	15.5	
maximum	0.224	0.4702	1.0388	0.6131	1291.0	9.717	99.5	37.1	68.7	
mean	0.1566	0.3102	0.6848	0.4483	283.0	4.659	56.1	17.7	30.5	
SD	0.03	0.07	0.13	0.07	175.59	1.97	20.14	7.26	10.48	
median	0.1508	0.2918	0.6593	0.439	258.0	4.049	50.6	14.9	28.9	

Table S4: Key statistic parameters calculated by determination of the degree of roughness of patients' right forearm. Skin relief replicas were analyzed via ODSCAD 6.3/PRIMOS 5.7 software.
